# Voluntary physical activity suppresses adipocyte hypertrophy through the activation of cGMP mediated pathway in a fructose-induced metabolic syndrome model in rat

**DOI:** 10.1007/s00394-025-03613-0

**Published:** 2025-02-15

**Authors:** Pınar Tayfur, Orkide Palabiyik, Burcu Meric, Ebru Tastekin, Selma Arzu Vardar

**Affiliations:** 1 Department of Physiology, Medical Faculty, Bahçeşehir University, İstanbul, Türkiye; 2 Institute of Health Sciences Trakya University, Edirne, Türkiye; 3 Department of Medical Services and Techniques, Trakya University Health Services Vocational College, Edirne, Türkiye; 4 Department of Pathology, Medical Faculty, Trakya University, Edirne, Türkiye; 5 Department of Physiology, Medical Faculty, Trakya University, Edirne, Türkiye

**Keywords:** Exercise, Fructose, Voluntary physical activity, Natriuretic peptide, White adipose tissue

## Abstract

**Purpose:**

A high-fructose diet is supposed to induce the so-called metabolic syndrome, associated with increased fat deposition in adipose tissue. Physical exercise may counteract the induction of the metabolic syndrome. The present study aims to investigate the effect of voluntary physical activity (VPA) on cGMP-mediated lipolysis in retroperitoneal adipose tissue in a metabolic syndrome model induced in rats by a high-fructose diet.

**Methods:**

Male Sprague-Dawley rats in control and fructose (F) groups had free access to either plain drinking water or a solution of 20% D-fructose, combined with a standard diet for 8 wk. Rats in the fructose + activity (F + A) group performed voluntary physical activity with a running wheel. Blood pressure, serum glucose, lipids and natriuretic peptide levels were measured on the last day of the feeding period. In retroperitoneal adipose tissue, cGMP, hormone-sensitive lipase (HSL), perilipin-1, aquaglyceroporin levels, and adipocyte diameter were analyzed.

**Results:**

Systolic blood pressure, glucose, and triacylglycerol were higher in the F groups compared to the control. The C-type natriuretic peptide was higher in the F group compared to the control. The cGMP level in retroperitoneal adipose tissue was higher in the F + A group than F group. Higher HSL and perilipin-1 levels were observed in the F + A group compared to the F and control groups. Adipocyte diameter was lower in the F + A group compared to the F group.

**Conclusion:**

Regular physical exercise triggers lipolytic effects in adipose tissue through cGMP, HSL, and perilipin-1-mediated pathway in fructose-induced metabolic syndrome model in rats, preventing the increase in adipocyte diameter.

## Introduction

High fructose consumption (sucrose or high-fructose corn syrup) plays a role in the development and progression of cardiac and metabolic dysfunction [1]. Experimental findings show fructose-induced hyperglycemia, insulin resistance, hypertension, as well as dyslipidemia and obesity in rats, and a metabolic syndrome model was created to evaluate these changes together [2]. The earliest metabolic alteration resulting from fructose consumption is postprandial hypertriglyceridemia [3]. In terms of visceral lipid accumulation, fructose consumption has been shown to cause non-alcoholic fatty liver disease [3]. It has been reported that non-alcoholic fatty liver disease may occur with the consumption of 10–30% liquid fructose in drinking water in animal models containing high fructose [4]. Moreover, fructose-rich diets are known to stimulate adipogenesis in white adipose tissue (WAT) [5]. Energy is stored in the form of triacylglycerol in WAT, and brown and beige adipose tissue can be distinguished by their distinct characteristics [6]. In rats, adipose tissue shows functional heterogeneity; retroperitoneal fat is mainly visceral adipose tissue [7]. Subcutaneous adipose tissue (SCAT) and WAT differ anatomically, cellularly, and metabolically. WAT has a greater capacity to generate free fatty acids and to uptake glucose than SCAT and is more sensitive to adrenergic stimulation. In the present study, the effect of a fructose-rich diet on rat adipocytes is investigated in WAT samples obtained from the retroperitoneal region. In the present study, the effect of a fructose-rich diet on rat adipocytes is investigated in WAT samples obtained from the retroperitoneal region.

Physical activity is considered to correct the high fructose diet-induced metabolic changes in adipose tissue [8]. Fructose infusion during exercise increases carbohydrate oxidation from fructose, lactate, and pyruvate in exercising muscles. Fructose infusion creates glucogenic effects in muscles and liver during the resting and recovery periods after exercise [9]. Additionally, exercise training performed during the period of high fructose intake plays a role in eliminating diastolic dysfunction caused by fructose overload [10]. Treadmill and swimming exercises are thought to improve metabolic effects such as dyslipidemia, hypertension, insulin resistance and glucose intolerance caused by a high-fructose diet in adipose tissue [1, 10]. The mechanisms underlying the metabolic effect of voluntary activity on running wheels as an experimental model is, however, incompletely understood. Voluntary physical activity in rodents leads to running behavior close to natural conditions without being exposed to factors such as electrical stimuli, and thus is comparable to physical activity in human life. Rodents exposed to a voluntary pattern of physical activity using running wheels generally perform exercise for long periods of time, traveling long distances at any time of the day [11].

A number of hormones such as catecholamines, insulin, and natriuretic peptides are effective regulators of lipolysis in WAT using different cellular signal pathways [12]. Studies on the lipolytic effect of exercise revealed the physiological functions of natriuretic peptides such as lipolysis activation, lipid oxidation, and mitochondrial respiration. Moreover, the cGMP-mediated signaling pathway was found to be actively involved in lipolysis in adipocytes [13]. We have shown in a previous study that exercise increases plasma C-type natriuretic peptide (CNP) levels in healthy human [14]. In addition, it is known that atrial natriuretic peptide (ANP) and B-type natriuretic peptide (BNP) cause lipolytic effects in adipose tissue [12]. However, it remains unclear whether CNP, which has important regulatory effects on lipid metabolism, glucose homeostasis and thermogenesis through cyclic guanosine monophosphate (cGMP) receptors, is able to affect WAT during a fructose-rich diet. The present study aimed to explore potential lipolytic effects of voluntary physical activity induced by different types of natriuretic peptides in a metabolic syndrome model of rats fed with a fructose-rich diet. For this purpose, the natriuretic peptide-mediated lipolytic effects of voluntary physical activity were investigated in retroperitoneal adipose tissue. Changes of natriuretic peptide levels in circulation resulting from voluntary physical activity were investigated in rats fed with drinking water containing 20% fructose for eight weeks.

Prior to energy conversion, triacylglycerol stored in adipocytes is hydrolyzed to non-esterified fatty acids and glycerol [12]. Triacylglycerol hydrolysis in adipocytes is controlled by three major cytoplasmic enzymes including adipose triglyceride lipase, hormone-sensitive lipase (HSL), and monoacylglycerol lipase. HSL has been identified a key enzyme in triacylglycerol hydrolysis, in WAT [15, 16]. Additionally, one of the most abundant proteins both on the surface and inside lipid droplets in adipocytes, i.e., perilipins, play a role by triggering lipolysis [17]. Perilipins are promoted by catecholamines or natriuretic peptides to generate lipolytic activation due to phosphorylation by protein kinase A and protein kinase C, respectively. Some of the lipolytic hormones, such as insulin and catecholamines, exert their effects through cAMP and protein A-mediated regulation, whereas natriuretic peptides are effective in stimulating lipolysis by the cGMP-dependent PKG signaling pathway. The hormonal lipolytic effects on adipocytes differ in the state of feeding, fasting, resting or exercise. According to the previous findings, natriuretic peptides may cause lipolytic effects through the activation of HSL and perilipin by affecting the cGMP-mediated signaling pathway [12]. However, the effects of voluntary physical activity-induced natriuretic peptide alterations on adipose tissue in high fructose fed rats have not been fully elucidated. The present study aimed to investigate changes of cGMP, HSL and perilipin-1 levels in retroperitoneal adipose tissue in rats fed with high fructose diet compared to rats performing voluntary physical activity in addition to a high-fructose diet.

In the present study, we also examined the adipose tissue levels of aquaglyceroporin (AQP), well-known transmembrane channels permitting adipocyte glycerol transport [18]. The content of AQP7, the first AQP identified in adipose tissue, is increased in skeletal muscle of obese mice [19]. AQP7 deficiency in adipose tissue is associated with triacylglycerol accumulation and obesity development. However, the effects of metabolic changes that may result from a fructose-rich diet on AQPs in adipocytes are not clear. Long-term elevation of insulin decreases the levels of AQP7 mRNA levels in adipocytes [20], although another study showed upregulation by insulin in adipocytes [21]. Additionally, elevation of AQP7 under short-term regulation of catecholamine and sympathetic activation strongly suggests an upregulation by physical exercise [20]. In addition to AQP7, AQP3 is a member of the aquaglyceroporin family [20]. A recent study found the expression of APQ3 in cell adipocyte culture [22], and AQP3 has been suggested to be a key modulator of adipocyte proliferation and differentiation [23]. Therefore, we included the measurement of AQP7 and AQP3 levels in the present study.

## Methods and materials

### Experimental model

Experiments were performed on 21 male Sprague-Dawley rats (220 ± 23 g body weight, 90 days of age at the start of the experiments) in the Experimental Animals Unit of Trakya University (Edirne, Türkiye). The animals were housed at controlled temperature of 21 ± 2 °C and humidity of 55%, and a 12-hour dark-light cycle (light from 07:00 to 19:00 h).

The rats stayed individually in plastic cages with free *ad libitum* access to purified water and standard laboratory chow (Optima Rat Chow-Bolu, Türkiye; see for chemical composition Table 1). Tap water was purified by passing through an automatic reverse osmosis water system. The duration of the experimental period was 8 weeks. The animals were randomly assigned to three groups. The rats in the control group (C, *n* = 7) received purified water during the experimental period. The fructose group (F, *n* = 7) received purified water enriched with fructose. Rats in the fructose plus activity group (F + A, *n* = 7) had access to the fructose-enriched drinking water and performed voluntary physical activity using the running wheel present in their cages.

**Table 1:** The ingredients of standard laboratory feed

Crude protein (%)	24.43
Crude cellulose (%)	3.08
Crude fat (%)	3.80
Starch %	31.53
Moisture %	12.72
Neutral detergent fiber %	9.02
Crude ash (%)	7.63
Lysine (%)	1.35
Methionine (%)	0.62
Calcium (%)	1.10
Phosphorus (%)	0.76
Sodium (%)	0.28
Vitamin A (I.U/kg)	36,000
Vitamin D3 (I.U/kg)	6500
Iodine (calcium iodate anhydrous) (mg/kg)	1.33
Cobalt (cobalt carbonate monohydrate) (mg/kg)	0.28
Copper (copper sulfate pentahydrate) (mg/kg)	16.66
Manganese (manganese oxide) (mg/kg)	156
Zinc (zinc oxide) (mg/kg)	130
Selenium (sodium selenite) (mg/kg)	0.35

The fructose-enriched solution was prepared daily by dissolving D-fructose (BIOMATIK, Cat. No: A2419, Cambridge, Ontario, Canada) in the drinking water to an end-concentration of 200 g/L to induce Metabolic Syndrome in group F and F + A. Water consumption (with or without fructose) was measured daily. Daily fructose consumption was calculated by subtracting the remaining drinking water volume from the total volume administered. The study was completed without any mortality in the experimental groups.

To determine whether rats meet the criteria for Metabolic Syndrome, the serum concentrations of triacylglycerol, total cholesterol, high-density lipoprotein (HDL-C) and low-density lipoprotein (LDL-C), glycerol, fatty acids, glucose and insulin were analyzed at the end of the 8-weeks feeding period. Body weight and naso-anal length were measured at the end of experimental period in gram and in mm, respectively. The Lee index [24], also a marker of Metabolic Syndrome, was calculated using the formula of the rats’ cube root of body weight (g)x10 divided by the naso-anal length (mm). Another marker of Metabolic Syndrome, i.e., blood pressure (BP) was measured on the first day and one day before the last day of the experimental period.

The night before the animals were sacrificed, they were fasted for 12–14 h from 19:00 for standardized serum measurements of metabolites, including serum lipids as markers of metabolic syndrome. The next morning between 07:00 and 11:00 h, the rats were anesthetized with intraperitoneal injection of ketamine (60 mg/kg) + xylazine (5 mg/kg). Then the thorax was opened, and a blood sample was collected directly from the left ventricular cavity with a 10-ml syringe and 22-gauge needle. The dose of ketamine used in this study (60 mg/kg) was below levels that would cause metabolic changes. A previous study reported that ketamine did not cause changes in plasma insulin concentrations and could affect lipoprotein lipase activity in adipose tissue only at high (120 and 140 mg/kg) concentrations [25].

Blood samples were allowed to clot at room temperature for 30 min. Thereafter, the clotted blood samples were centrifuged at 3000 rpm for 15 min at 4 °C to obtain serum. The serum samples were stored at -80℃ until analysis. After collection of blood, the animals were rapidly sacrificed by exsanguination and tissue samples of the intraperitoneal tissue were obtained. Half of the samples were placed in liquid nitrogen and transferred into cryotubes and stored at -80 °C for protein analysis using the ELISA method. The other half of the retroperitoneal adipose tissue samples were placed in 10% formalin solution for histological/histochemical analysis. Thereafter, the heart and liver were extirpated and weighted. Subsequently, the right and left ventricle of the heart were obtained, and their weight measured. Heart weight (mg)/tibia length (mm) ratios were calculated to assess exercise-induced cardiac hypertrophy, if any [26]. The experimental protocol was approved by the Trakya University Animal Experiments Local Ethics Committee (TUHADYEK-2018/34).

### Voluntary physical activity

The rats of group F + A were housed in plastic cages individually with free access to a stainless-steel running wheel as described previously [27]. The running wheel was mounted in each cage and a device recording the frequency of rotation were used, allowing for the measurement of the daily running distance. Similar to previous studies [28], the rotating wheels were 31.5 cm in diameter and 10 cm in width, and were placed in cages with a wheel base of 7 cm height above the floor of the cage and a circumference of 1081 cm. The running wheels were removable and cleanable. The rats of the F + A group were kept in cages with this kind of running wheel two weeks before the start of the experiment to familiarize the animals with the device. Based on our previous experiences, 2000 m/day was taken as the minimum average running activity required to qualify the rat as physically active [29].

### Blood pressure and heart rate measurements

Blood pressure (BP) and heart rate (HR) was determined weekly with the tail-cuff plethysmography method in conscious rats using a tail sensor and a noninvasive blood pressure analyzer (MAY NIBP250 Commat-Türkiye). Prior to blood pressure measurements, each rat was placed in a heat cabinet with a restrainer, thereafter the ambient temperature of the cabinet was slowly increased to 32–34 °C. After 30 min to allow the rats to adapt and calm down, the warmth-induced dilated tail artery was ready for the measurements. A cuff was placed and inflated on the tail and the cuff was released several times to condition the rat for the procedure. BP and HR were measured five times by analyzing peak-by-peak pulses to acquire an average value. The measurements were performed at one-minute intervals, the highest and lowest measurements were excluded to obtain reliable blood pressure measurements, and the remaining three measurements were used to calculate the average systolic blood pressure (SBP), diastolic blood pressure (DBP), similar to a previous study [30].

### Serum lipid and glucose concentrations

Serum triacylglycerol, total cholesterol, HDL-C, and LDL-C levels were measured by an enzymatic method (Abbot Architect c16, USA). The glycerol concentration in serum samples was determined using the Glycerol Colorimetric Assay Kit (BioVision Cat. No: K630-100, CA, USA) following the manufacturer’s instructions. The fatty acid concentration in serum samples was determined using the Free Fatty Acid Quantification Colorimetric Kit (BioVision Cat. No: K612-100, CA, USA) according to the manufacturer’s instructions. Glycerol and free fatty acid absorbances were measured using a spectrophotometer at 570 nm wavelength. A standard curve was constructed for each plate, and values were presented as mmol/L. The analyses of glycerol and free fatty acid concentrations were performed in duplicate.

Serum glucose levels were measured by an enzymatic method (Abbot Architect c16, USA), and insulin levels were determined by the enzyme-linked immunosorbent assay (ELISA) method (BT LAB Cat. No: E0707Ra, Shanghai, China). The Insulin resistance (HOMA-IR: Homeostatic Model Assessment for Insulin Resistance) value was calculated by the formula of HOMA-IR: serum insulin (mU/L) times blood glucose (mmol/L) divided by 14.5 [31].

### Serum ANP, BNP and CNP concentration

ANP (Cat. No: E0642Ra), NT-proANP (Cat. No: E1810Ra), BNP (Cat. No: E0475Ra), NT-proBNP (Cat. No: E0067Ra), CNP (Cat. No: E0055Ra) and NT-proCNP (Cat. No: E1811Ra) concentrations were measured by ELISA in duplicate (BT LAB, Shanghai, China). Detection ranges of the ELISA kits used in these analyses were 5 ng/L-1500 ng/L for ANP, 7 ng/L-1500 ng/L for NT-proANP, 1 ng/L-400 ng/L for BNP, 5 ng/L-2000 ng/L for NT-proBNP, 0.05 ng/mL-20 ng/mL for CNP, and 3 ng/L-900 ng/L for NT-proCNP. Intra-assay variation was less than 8% and inter-assay variation was less than 10%.

### Tissue homogenization and measurement of protein content

Frozen retroperitoneal adipose tissue samples were minced with a scalpel and weighed on a precision scale. Then, 300 µL of cold homogenization buffer (Sigma-Aldrich, St. Louis, MO, USA) was added (10 mM phosphate-buffered saline [PBS], pH: 7.4, 500 µL/L Triton X-100) to approximately 300 mg of the frozen, minced adipose tissue. Thereafter, the samples were homogenized with an ultrasonic homogenizer for 10 s in an ice-cold environment. The homogenates were centrifuged at 3000 rpm for 10 min at 4 °C. The uppermost fat layer was removed from each tube and discarded. The remaining homogenate was maintained. Subsequently, 400 µL of ethanol: chloroform mixture (2:3) was added per 0.8 mL homogenate. The mixture was first vortexed in a vortex mixer and then centrifuged at 10,000 x g rotation speed at + 4 °C for 30 min. After centrifugation, two lipid layers were formed on top and bottom of the test tube with a liquid layer in between. The liquid layer was collected as ‘supernatant’. Supernatant processing was carried out using the method described by Alver et al. [32]. The protein concentration of supernatants was measured with the BCA Protein Assay Kit (SMART BCA Protein Assay Cat. No: 21071, MA, USA). The resulting supernatants were used in ELISA analyses.

### Biochemical analysis of homogenates of retroperitoneal adipose tissue

In the present study, we determined the tissue content of cGMP (Cat. No: E0297Ra) being the second messenger stimulated by natriuretic peptides, the key lipolytic enzyme HSL (Cat. No: E0922Ra), and of AQP7 (Cat. No: E1421Ra) and AQP3 (Cat. No: E0567Ra), being proteins transporting glycerol as well as water in retroperitoneal adipose tissue, using an ELISA kit (BT LAB, Shanghai, China) according to the manufacturer’s instructions.

### Histological analysis of adipose tissue

Retroperitoneal adipose tissue, fixated in 10% buffered formalin solution for 24 h, were submerged in paraffin blocks and sectioned to 4 μm thickness. Deparaffinization was carried out by keeping the slices in a 70^o^C oven for 1 h and subsequently in xylene at room temperature for 45 min. Slices were passed through graded ethanol solutions (ethanol content: 90-80-70%) and stained with hematoxylin-eosin (H&E) for the histo-morphometric examination of adipocytes under the light microscope (Nikon Eclipse E600). Tissue slices randomly selected per animal were embedded in paraffin blocks and each section was examined at X10 magnification using a light microscope. Cell diameter was determined using 30 adipocytes per section, three sections per animal, and seven animals per group. Dominant adipocyte population was selected, analyzed, and measured in each section.

For immunohistochemical staining of HSL and perilipin 1 (PLIN-1) levels in retroperitoneal adipose tissue, 4 μm thick sections prepared from formalin-fixed and paraffin-submerged tissues were placed on electrostatically charged slides and dried at 70 °C for at least 1 h. The immunohistochemical staining process was performed with a fully automatic immunohistochemical staining device (Ventana BenchMark Ultra IHC/ISH System, Tucson, AZ, USA) and antigen retrieval was performed. Anti-HSL antibody (mouse monoclonal antibody, 1:50 dilution, Cat. No: sc-74489, Santa Cruz Biotechnology, USA) and anti-perilipin 1 antibody (rabbit polyclonal antibody, 1:50 dilution, Cat. No: bs-10779R, Bioss Antibodies, USA) were applied to the adipose tissue slices, separately. “The entire immunohistochemical staining process, including deparaffinization, rehydration and antigen retrieval is performed by CC1 (prediluted; pH 8.0) antigen retrieval solution (Ventana Medical Systems, Roche Group, Tucson, AZ, USA), performed on the BenchMark ULTRA automated slide stainer (Ventana) for 64 minutes at 100°C (default temperature on ULTRA). The human adrenal gland served as positive control for HSL, and adipose tissue for PLIN-1. Positivity was determined by membranous staining for PLIN-1. Both for HSL and PLIN-1, at least five fields selected randomly were examined at 40X magnification and the results were expressed as percent staining.

Each case was scanned in at least five randomly selected fields at 40x magnification and reported as staining percentages. The percentage of immunopositive cells was categorized as follows: score 1: 0–5%, score 2: 5.1–50%, score 3: 50.1–80%, score 4: 80.1–100%. Additionally, scoring was performed based on the intensity of immunohistochemical staining. Staining intensity was classified as follows: score 0: negative, score 1: weak, score 2: moderate, score 3: strong. The staining score for each sample was obtained by multiplying the staining extent score by the staining intensity score [33]. All these assessments were conducted by a researcher who is an experienced pathologist.

### Statistical analysis

All values ​​are expressed as mean ± standard deviation (SD). Data were analyzed using the SPSS (Statistical Package for the Social Sciences) software. The Shapiro-Wilk test was used to check the normality of data distribution and the differences between variances. One-way ANOVA test and Tukey’s test were utilized to compare the variables showing normal distribution between the groups. The Kruskal-Wallis test and Mann-Whitney-U test were employed to compare the variables lacking a normal distribution. The Wilcoxon signed-rank test was used to compare activity levels between study weeks. A p-value ≤ 0.05 was considered statistically significant.

## Results

### Body weight measurements

Body weight was found to be comparable across the groups at the beginning of the experimental period (C: 217 ± 19 g, F: 224 ± 29 g, and F + A: 221 ± 22 g) and at the end of week 8 (C: 390 ± 25 g, F: 389 ± 35 g and F + A: 389 ± 18 g) (*p* = 0.838 and *p* = 0.996, respectively). However, the Lee index values ​​determined at the end of the experiment were significantly lower in both the F and F + A groups compared to the C group (*p* < 0.001 and *p* = 0.019, respectively; Table 2).

**Table 2:** Body weight, Lee index and tissue weight data

Parameters/Groups	Control	Fructose	Fructose + activity	*P*
Body weight (g)				
Initial weight (g)	217 ± 19	224 ± 29	221 ± 23	0.838
Final weight (g)	390 ± 25	389 ± 35	389 ± 17	0.996
Naso-anal length (mm)	22.3 ± 0.7	23.5 ± 0.6*	23.0 ± 0.5	**0.003**
Lee index (g/mm)	0.328	0.310*	0.318*	**< 0.001**
Heart weight (g)	1.38 ± 0.14	1.32 ± 0.09	1.46 ± 0.09	0.081
Tibia length	4.5 ± 0.2	5.1 ± 0.2*	4.9 ± 0.16*	**< 0.001**
Heart weight/Tibia length (mg/mm)	30.73 ± 3.57	25.78 ± 2.05	29.59 ± 1.73#	**0.003**
Left ventricle weight (g)	0.377 ± 0.118	0.382 ± 0.079	0.535 ± 0.048*#	**0.004**
LV weight/ BW (g/kg BW)	0.972 ± 0.319	0.990 ± 0.25	1.370 ± 0.14*#	**0.010**
Right ventricle weight (g)	0.218 ± 0.087	0.179 ± 0.055	0.160 ± 0.019	0.209
Liver weight (g)	15.77 ± 2.53	16.68 ± 1.58	17.11 ± 1.29	0.405

Values are expressed as mean ± SD. *p < 0.05 vs. Control. #*p* < 0.05 vs. Fructose. Left ventricle weight/ Body weight (LV weight/ BW)

### Voluntary running wheel activity

All animals in the F + A group completed the exercise period without any injury during the experiment. Based on a previous study, 2000 m/day was determined as the minimum average running activity required for a rat to be qualified as physically active [28]. In the present study, the average running distance was approximately 2200 m/day during Week 1. Running distances reached 5500 and 5800 m/day in Weeks 2 and 3, respectively. The rats then maintained their average running distance of varying from 2000 to 12,000 m/day until the end of the 8-week-long period. The running wheel activities of the rats showed an increase up to four weeks, followed by a decreasing trend after the fourth week (*p* < 0.05). The physical activity records showed that the mean running distance of the F + A group was 5055 ± 1355 m/day over the total eight-week experimental period.

### Blood pressure and heart rate values

Systolic, diastolic, mean arterial blood pressure and heart rate values were similar before the feeding period in all groups (Day 1). The end of the feeding period (Week 8), systolic blood pressures of the F and F + A groups were significantly higher compared to the C group (*p* = 0.003 and *p* = 0.035, respectively). No significant difference was shown between the groups in diastolic blood pressure, mean arterial blood pressure and heart rate values measured at 8 weeks (Table 3).

**Table 3:** Hemodynamic findings, baseline and at week 8

Parameters/Groups	Control	Fructose	Fructose + activity	*P*
SBP (mmHg)				
Baseline	128 ± 2	129 ± 3	129 ± 3	0.683
Week 8	127 ± 5	138 ± 8*	133 ± 7*	**0.006**
DBP (mmHg)				
Baseline	109 ± 4	111 ± 3	110 ± 4	0.544
Week 8	107 ± 8	113 ± 10	112 ± 4	0.333
MAP (mmHg)				
Baseline	115 ± 3	117 ± 3	116 ± 3	0.483
Week 8	114 ± 7	122 ± 9	119 ± 5	0.135
Heart rate (beats/min)				
Baseline	396 ± 25	388 ± 21	396 ± 28	0.756
Week 8	334 ± 14	350 ± 10	345 ± 15	0.094

Values are expressed as mean ± SD. *p < 0.05 vs. Control. Systolic blood pressure (SBP), diastolic blood pressure (DBP), mean blood pressure (MAP) and heart rate

### Consumption of drinking fluid and fructose

Fluid intake of the F + A group (78 ± 11 mL/day) was significantly higher than in the C group (56 ± 4 mL/day) and the F group (64 ± 9 mL/day), (*p* = 0.001 and *p* = 0.023, respectively).

The average fructose consumption in the F + A group amounted to 15.6 g fructose per day. In the F group the average consumption was 12.8 g per day (*p* = 0.048).

### Lipid-related metabolic parameters and glucose and insulin concentrations in serum

Triacylglycerol levels ​​in the F and F + A groups were significantly higher compared to the C group (*p* = 0.001 and *p* < 0.001, respectively); difference between group F and FA was not significant (*p* > 0.05). Total cholesterol, HDL-C and LDL-C, glycerol and free fatty acid levels were similar in all groups. Glucose levels ​​in F and F + A groups were significantly higher compared to the C group (*p* = 0.003 and *p* = 0.004, respectively). No difference in glucose concentration was observed between group F and F + A. While no significant difference was found in insulin values between the F and C group (*p* = 0.142), a significant decrease was observed in the F + A group compared to the C group and F group (*p* < 0.001 and *p* < 0.001, respectively). The HOMA-IR values in the F group were found to be higher than that in group C (*p* = 0.042). However, a significant decrease was observed in HOMA-IR values in F + A group compared to C group and F group (*p* = 0.004 and *p* = 0.002, respectively; Table 4).

**Table 4:** Serum metabolic parameters related to metabolic syndrome

Parameters/Groups	Control	Fructose	Fructose + activity	*P*
Triacylglycerol (mg/dL)	86 ± 31	185 ± 27*	210 ± 64*	**< 0.001**
Total cholesterol (mg/dL)	77 ± 11	73 ± 13	75 ± 5	0.764
HDL-C (mg/dL)	44 ± 6	40 ± 7	43 ± 5	0.507
LDL-C (mg/dL)	24 ± 4	21 ± 1	24 ± 3	0.388
Glycerol (mmol/L)	1.50 ± 0.2	1.83 ± 0.2	1.68 ± 0.5	0.189
Fatty acid (mmol/L)	4.67 ± 0.9	6.16 ± 2.2	4.54 ± 1.2	0.133
Fasting glucose (mmol/L)	13.93 ± 1.7	24.36 ± 3.49*	23.03 ± 3.27*	**0.003**
Fasting insulin (ng/mL)	0.87 ± 0.2	0.72 ± 0.2	0.19 ± 0.1*#	**< 0.001**
HOMA-IR index	0.82 ± 0.09	1.21 ± 0.46*	0.32 ± 0.15*#	**0.001**

Values are expressed as mean ± SD. *p < 0.05 vs. Control; #*p* < 0.05 vs. Fructose. High-density lipoprotein (HDL-C) and low-density lipoprotein (LDL-C), homeostatic model assessment for insulin resistance (HOMA-IR index)

### Cardiac and liver tissue weight

The left ventricular weight in the F + A group was significantly higher than in the C and F groups (*p* = 0.008 and *p* = 0.008, respectively). In terms of left ventricular weight/body weight ratios, the F + A group had higher values than the C group and F group (*p* = 0.018 and *p* = 0.020, respectively). The heart weight/tibia length (HW/TL) ratio was significantly higher in the F + A group than in the F group (*p* = 0.004; Table 2). There was no significant difference between the groups in right ventricular weight (*p* = 0.209) and liver weight (*p* = 0.405; Table 2).

### Serum ANP, BNP and CNP concentration

**Fig. 1:**
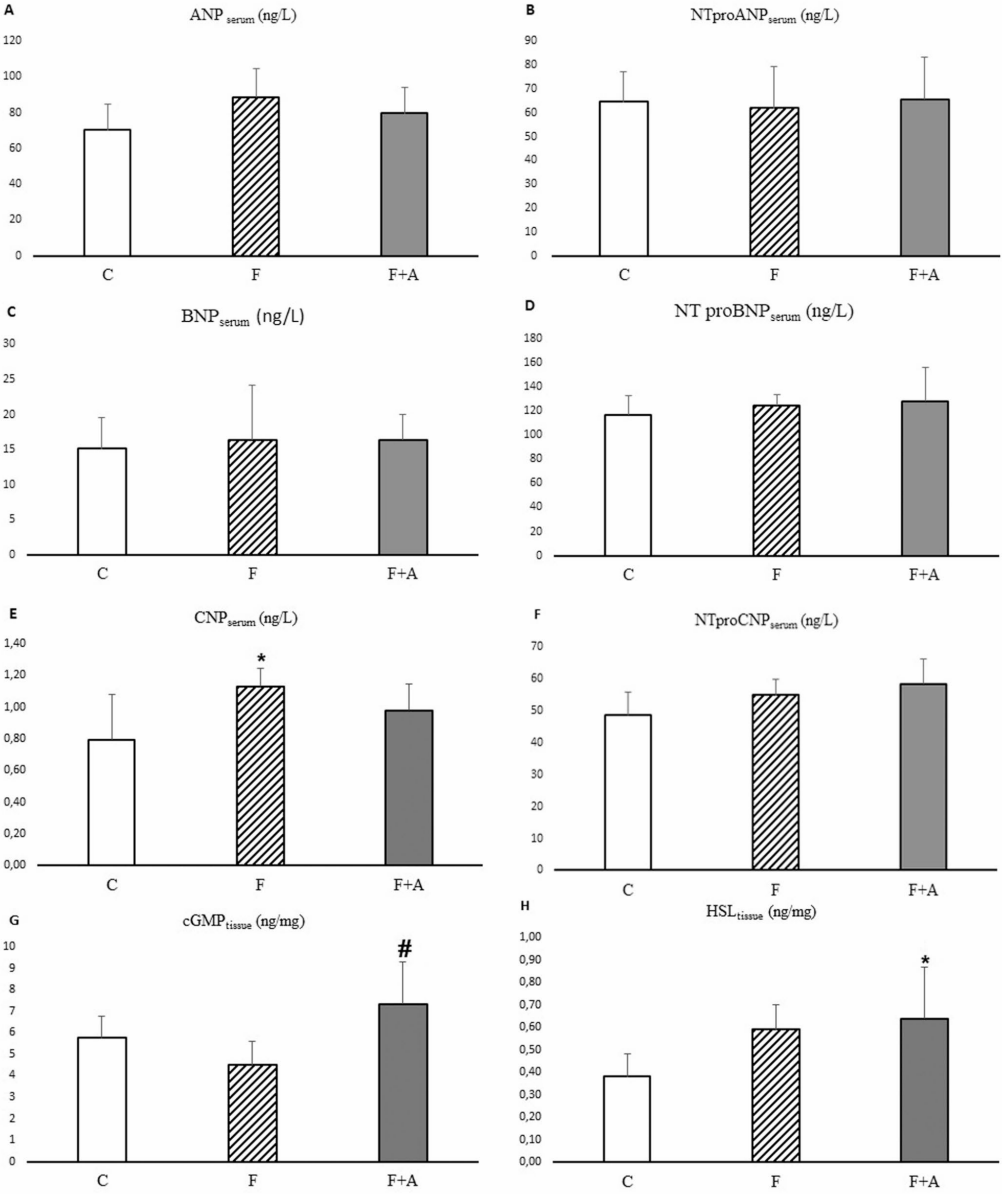
Natriuretic peptide concentration in serum and lipolysis parameters in retroperitoneal adipose tissue. A-ANP: Atrial natriuretic peptide, B- NTproANP: N-terminal pro-atrial natriuretic peptide, C-BNP: B-type natriuretic peptide, D-NTproBNP: N terminal pro B-type natriuretic peptide, E-CNP: C-type natriuretic peptide (*0.016), F-NTproCNP: N-terminal pro C-type natriuretic peptide. G-cGMP: Cyclic guanosine monophosphate (#0.004), H-HSL: Hormone-sensitive Lipase (*0.036). Values are expressed as mean ± SD. *p < 0.05 vs. Control; #*p* < 0.05 vs. Fructose

CNP serum levels in the F group were significantly higher than the group C (*p* = 0.012). Serum ANP, NT-proANP, BNP, NT-proBNP and NT-proCNP concentrations were not significantly different among the three groups (Fig. 1).

### cGMP, AQP3 and AQP7 levels and HSL content in retroperitoneal adipose tissue

In our study, cGMP levels in the F + A group were found to be significantly higher than in group F (*p* = 0.003). However, no statistically significant difference was found between the F + A and C group (*p* = 0.116). A significantly higher HSL content was observed in the F + A group compared to group C (*p* = 0.036; Fig. 1). No significant difference was found when F + A and F groups were compared (*p* = 0.844). There was no difference between the groups in terms of AQP3 and AQP7 levels in retroperitoneal adipose tissue (*p* = 0.451 and *p* = 0.931, respectively).

### Histological values and immunoreactivity ​​in retroperitoneal adipose tissue

**Fig. 2:**
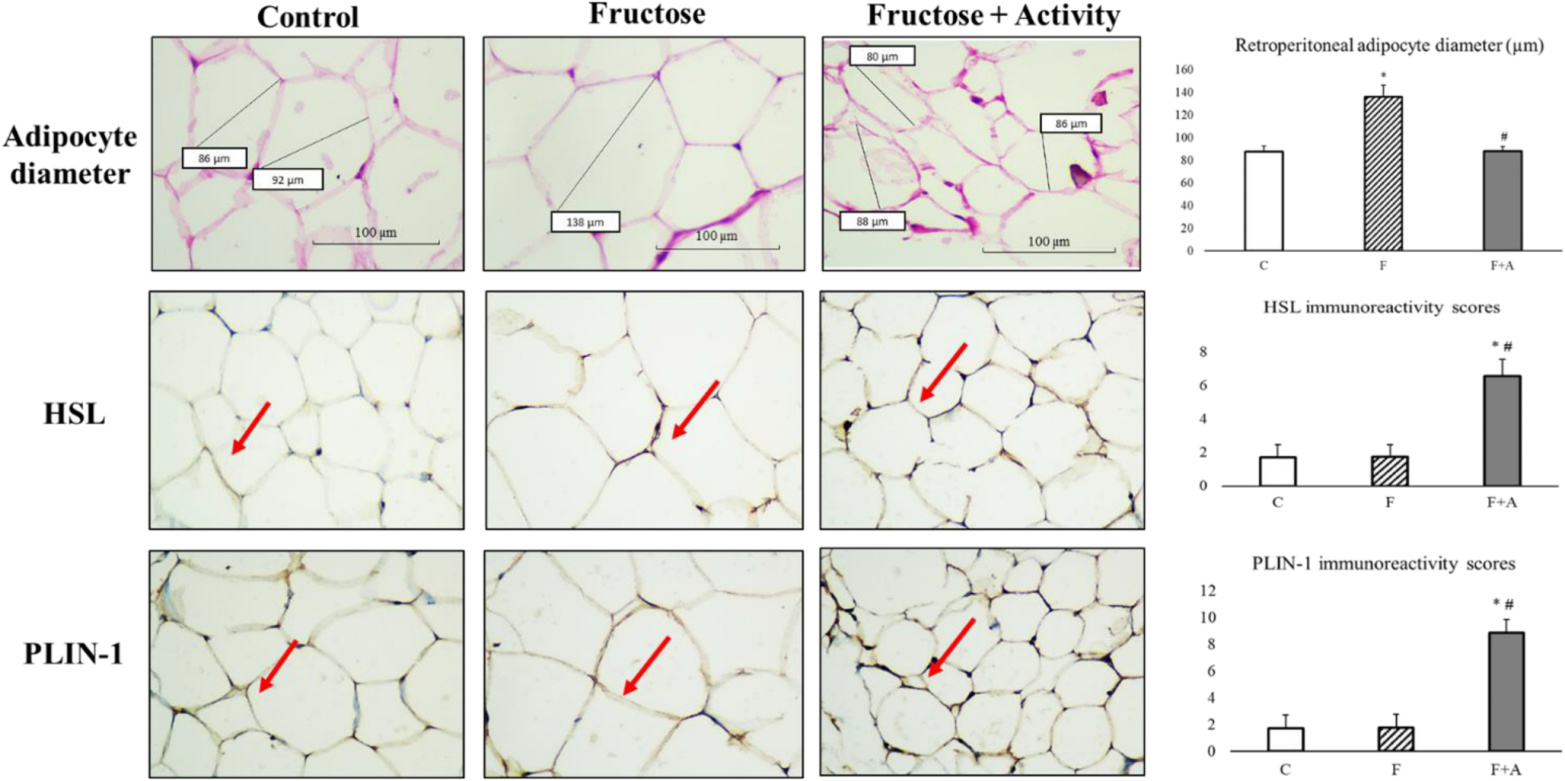
Hematoxylin and eosin-stained sections of adipocytes (x40) and the comparison of adipocyte diameter of the groups. HSL and PLIN-1 immunohistochemical staining and the immunoreactivity scores in retroperitoneal adipose tissues sections in C group, F group, and F + A group C: Control, F: fructose, F + A: Fructose + activity. Scale bar: 100 μm. Values are expressed as mean ± SD. *p < 0.05 vs. Control; #*p* < 0.05 vs. Fructose

A significant difference was found between the groups in terms of adipocyte diameters in retroperitoneal adipose tissue samples examined under light microscopy (*p* < 0.001). Adipocyte diameters (136 ± 10 μm) in F group were significantly higher compared to the C group (87 ± 5 μm) (*p* < 0.001). Adipocyte diameters in F + A group (88 ± 4 μm) were found to be significantly decreased compared to the F group (Fig. 2).

Mild staining was observed in HSL immunoreactivity in retroperitoneal adipose tissue of rats in C and F groups, while diffuse and strong staining was observed in F + A group (Fig. 2; red arrow). A significant increase was observed in HSL levels in the retroperitoneal adipose tissue sections of F + A group compared to C and F group (*p* = 0.001 and *p* = 0.001, respectively). When retroperitoneal adipose tissue sections of the rats were immunohistochemically stained with PLIN-1 antibody, mild staining was observed in groups C and F, whereas diffuse and strong staining was observed in F + A group (Fig. 2; red arrow). A significant increase was observed in PLIN-1 levels based on intensity of staining in the retroperitoneal adipose tissue sections of group F + A compared to group C and group F (*p* = 0.001 and *p* < 0.001, respectively).

## Discussion

The findings of the present study showed a significant increase in serum CNP level and adipocyte size in male rats in the metabolic syndrome model created using high fructose feeding for eight weeks. According to the findings of the present study, cGMP pathway was observed to be involved in the cellular lipolytic effect of voluntary physical activity in retroperitoneal adipose tissue.

Establishing a metabolic syndrome model in rodents requires at least three criteria [34]. In this study, an increase was observed in systolic blood pressure, serum triacylglycerol content, glucose levels and insulin resistance after eight weeks of high fructose consumption. Therefore, an experimental metabolic syndrome model induced by high-fructose-fed rats was successfully established. In addition to these parameters, obesity development can be considered as another parameter to be investigated in metabolic syndrome models. However, according to the Lee index data, which is reported as the appropriate marker to evaluate obesity [10], no evidence of obesity development in rats was obtained when subjected to a high-fructose diet. We observed that body weight was similar in all groups whereas naso-anal length and tibia length were higher in F group than control. According to a previous study, a fructose-rich diet for eight weeks shows positive effect on bone mass [35]. Considering total body mass as an obesity marker, it can be concluded that in this study with an 8-week period to fructose-exposure no signs of overall pathological obesity are present.

There is controversial data in the literature on whether weight gain is observed in metabolic syndrome models associated with high fructose consumption [34]. While some previous studies have reported increased weight [36] and some others reported decreased weight [37].

There are also studies reporting no weight change in metabolic syndrome model [38]. Morvan et al. [38] reported that body weight did not change in animals given fructose in drinking water for eight weeks, similar to our study. In addition, it has been reported that Sprague-Dawley rat strains do not gain excess weight on fructose diets despite becoming leptin-resistant [39]. However, an increase in visceral adipose tissue weight was reported compared to the control; fructose has been reported to stimulate the differentiation of adipocytes in retroperitoneal WAT in an in vitro study [40]. Although we observed no significant change regarding weight gain with the high fructose feeding used in our study, the increase in adipocyte diameters can be considered as an indication of adipocyte hypertrophy in WAT resulting from a high fructose feeding.

It is known that the increased intracellular accumulation of triacylglycerol in the adipocytes causes an increase in the adipocyte volume [41]. In our study, the increase in serum triacylglycerol levels in the high-fructose-fed groups may be a factor in favor of adipocyte hypertrophy. In another study, rats fed with a-high fructose, an increased number of subcutaneous abdominal adipocytes has been reported as well as an increased diameter of intra-abdominal adipocytes [42]. It has also been reported that adipocyte size is associated with resistance to insulin action [42] and that, as another factor, exercise diminishes the adipocyte size (36). The higher values in insulin resistance detected in the fructose group in comparison to fructose + activity group in our study may be considered as an additional factor involved in the development of adipocyte hypertrophy.

To understand the effect of voluntary physical activity, the group performed voluntary physical activity was also fed high fructose. Our findings showed that high fructose consumption resulted in an increase in insulin resistance and consequent increase in serum glucose level in F group compared to C group. However, blood pressure, triacylglycerol and glucose levels at the end of the feeding period did not differ in high fructose-fed rats with voluntary physical activity than without voluntary physical activity. A previous study by Morishima-Yamato et al. [43] showed that male rats performing voluntary running wheel activity had lower body weight, less visceral adipose tissue and decreased circulating insulin levels compared to the controls. It has been reported that the regulation of insulin and glucose tolerance by voluntary physical activity may be related to increasing glucose transporter type-4 (GLUT4) levels and, hence, exercise-induced improvement can be achieved [44]. According to the findings of this study, voluntary physical activity may not seem to be effective in reducing body weight, serum glucose and triacylglycerol levels in the high-fructose-fed rats. Although it is unclear whether voluntary physical activity affects caloric or water intake in high fructose fed rats, one of the reasons of this experimental outcome may be that the daily intake of fructose-rich fluid in the F + A group (78 ± 11 mL∕day) was found to be significantly higher than in the F group (64 ± 9 mL∕day).

In the present study, AQP7 and AQP3 levels were examined in retroperitoneal adipose tissue of rats performing voluntary activity and no significant change was found between the groups. AQP7 and AQP3 as water and glycerol channels, respectively, are suggested to play a regulatory role in energy metabolism by affecting insulin secretion in the body. In a previous study, we observed that exercise increased the expression of AQP7 in cardiac tissue [45]. Based on the present findings, we conclude that in adipose tissue a high-fructose diet does not affect the content of these two aquaporins.

In the present study, higher serum CNP level was observed in high-fructose-fed rats in comparison to control rats. Previous studies have reported that CNP regulates food intake and energy expenditure [46]. Moreover, it has been shown that CNP, overexpressed in endothelial cells of visceral adipose tissue, increases thermogenesis in brown adipose tissue, reduces adipocyte hypertrophy, decreases WAT weight, and inhibits fatty liver development [47]. CNP has also been reported to regulate blood glucose levels, reduce insulin resistance, prevent adipose tissue expansion, and stimulate the expression of thermogenic markers [47–49]. Our findings may indicate that higher CNP levels in the serum of the high-fructose-fed rats do not play a considerable role in serum triacylglycerol, glucose levels and adipocyte diameter to prevent the development of obesity triggered by a short time period of high-fructose feeding. Although the CNP level of F + A group was not different from that in the C and F group in this study, increasing tendency in F group without significant elevation in F + A group suggests that voluntary physical activity may be a factor that reduces serum CNP levels in high-fructose-fed rats.

It has been shown that catecholamine and glucagon-mediated cAMP pathway and insulin-mediated phosphodiesterase-3 activation are involved in the regulation of lipolysis in adipocytes [50]. In addition, natriuretic peptides exert their effect through a cGMP-dependent pathway. Sengenes et al. [51] reported that ANP exerts lipolytic effects in both human adipose tissue and isolated adipose cells in vitro, and that these effects were associated with a significant increase in intracellular cGMP levels. In our study, we observed higher CNP levels without any alteration in cGMP levels in the fructose-fed group. Although there was no significant change in serum natriuretic peptide levels in fructose-fed rats that performed voluntary physical activity, we observed increased cGMP levels in F + A group (Table 3). These findings suggest that the increase in cGMP in the F + A group may occur due to factors other than CNP. Using micro-dialysis experiments, Sengenes et al. [51] confirmed the in vivo lipolytic effect of ANP in the abdominal subcutaneous tissue of healthy young males. They also found that circulating concentrations of ANP was lipolytic in human [51]. Although there was no significant increase in serum ANP levels compared to the controls in the present study, it can be speculated that the increase in cGMP levels might be caused by enhanced adipocyte sensitivity towards circulating ANP levels.

After binding to their receptors on the adipose tissue membrane, natriuretic peptide hormones increase intracellular cGMP levels by activating guanylate cyclase, and cause phosphorylation of hormone-sensitive lipase (HSL) as well as the lipid droplet-related surface protein, perilipin-1 (PLIN-1) in the cytoplasm of adipocytes [52]. Perilipins, present on the surface of intracellular lipid droplets, exert their lipolytic effect at this location, resulting in the release of fatty acids into the plasma [17]. In the present study, mild staining for HSL and PLIN-1 was observed in retroperitoneal adipose tissue samples in F group and C group whereas strong staining was observed in F + A group, suggesting that the cGMP-mediated lipolytic effect is a result of voluntary physical activity. Therefore, HSL and PLIN-1 involved in the lipolytic process are likely target proteins of physical activity in the non-pharmacological treatment of metabolic syndrome and obesity. Although adipose tissue HSL protein level was found to be high in the F + A group in this study, it was not significantly different from the F group, suggesting that a more detailed examination of protein expression is warranted.

Considering the importance of PLIN-1 and HSL phosphorylation in evaluating lipolysis, phosphorylated forms of these parameters that were not examined in this study can be considered as a limitation. The other limitation of this study is that fructose consumption was variable across the fructose groups because it allowed the animals to drink fluids and consume fructose freely. This variability can make it difficult to interpret certain results, as both exercise and different doses of fructose intake may have influenced the observed effects. Despite the exercise group consuming more fructose, some of the findings improved in this study. However, if the fructose dosage had been matched, it would have been more possible to show whether other changes were also improved by exercise. The present study was also limited by its lack of a control group consisting of animals with free access to voluntary physical activity and received purified water without added fructose during the experimental period. Therefore, comparisons between groups were obtained from the three groups.

In conclusion, this study showed increased serum CNP levels and adipocyte hypertrophy in retroperitoneal adipose tissue in an experimental metabolic syndrome model in rats exposed to high fructose feeding. It has been shown that voluntary physical activity may suppress the fructose-induced increase in serum CNP levels. Although in the present study regular daily exercise did not cause weight loss, a lower blood pressure, and a decline in serum triacylglycerol and glucose levels in rats fed on a high-fructose diet, the physical exercise was found to be associated with a decline in diameter of retroperitoneal adipocytes, an effect most likely to be mediated through cGMP-dependent pathways.
